# Physiotherapy Rehabilitation Strategies for Post-operative Trimalleolar Ankle Fracture: A Case Report

**DOI:** 10.7759/cureus.29716

**Published:** 2022-09-28

**Authors:** Pranali S Fokmare, Pooja Dhage

**Affiliations:** 1 Musculoskeletal Physiotherapy, Ravi Nair Physiotherapy College, Datta Meghe Institute of Medical Sciences, Wardha, IND

**Keywords:** case report, ultrasound, triamalleolar ankle fracture, mulligan’s mobilization, physiotherapy rehabilitation, pain

## Abstract

The trimalleolar fracture of the ankle is the least common type of fracture among ankle fractures. This type of fracture occurs due to high-energy trauma and is seen mostly in females than males. In this case report, we present a 52-year-old female patient who met with a road accident 15 months before presentation. She was brought to the hospital for investigation and was diagnosed with a trimalleolar ankle fracture. She was managed with open reduction and internal fixation using rush nailing and canulated cancellous screws. There was pus discharge from the suture site post-operatively. Due to this, there was a delay in the weight-bearing phase. Medications were given for relief. Ten months post the operation she saw pus discharge from the scar site, she was re-operated for implant removal and physiotherapy was advised. Due to fear of pain, she was not bearing weight on that limb. This prolonged non-weight-bearing phase caused muscle wasting of the right leg, and reduced ankle dorsiflexion range and strength. The patient attended physiotherapy treatment. A Mulligan’s movement with mobilization was prescribed to increase the range of ankle dorsiflexion, and ultrasound was used to improve scar mobility. Strength training of lower limb muscles, proprioception training, and gait training was given to the patient. The treatment showed improvement in the range and strength of the lower limb.

## Introduction

Ankle fractures are the most typical type of fracture in the lower limbs. These fractures could be unimalleolar, bimalleolar, or trimalleolar, depending on the number of malleoli involved. A bimalleolar fracture plus a posterior malleolar fracture make up the trimalleolar ankle fracture (TAF), a kind of complex ankle fracture. It is the least common kind and only appears in 7% of all ankle fractures [1]. High energy trauma is to blame for these fractures [2]. Females experience incidence at a 2:1 higher rate than males do. One study found that females had a higher prevalence of trimalleolar fractures, with 77.78% of participants being female. This might be caused by osteopenia or osteoporosis, which may be found in this vulnerable patient group [3]. The reduction and treatment of this kind of fracture remain a major challenge in the medical field. Following treatment, patients usually encountered some kind of sequelae, like joint stiffness, traumatic arthritis, walking instability, and joint deformity [4]. One of the techniques available in physiotherapy for regaining range of motion is joint mobilization. The Mulligan idea of mobilization with movement (MWM), a form of manual therapy, involves the therapist using a gliding force while the patient performs an active movement [5]. In order to restore the lower limb's natural supporting function, the applied exercise therapy's goal is to repair the ankle joint as functionally as possible [2]. Rehabilitation begins after surgery or the removal of a plaster cast. The combination of restricted active and passive range of motion, decreased muscle strength, altered proprioception, and pain as a result of the fracture and treatment necessitates a specialized rehabilitation program that includes exercises for ankle mobility, strengthening, weight-bearing, and balancing [6].

## Case presentation

Patient information

This is a case of a 52-year-old female patient with right-handed dominance, a teacher by occupation at a school. She met with a road accident and suffered injuries, her right ankle was twisted. She was taken unconscious to the hospital where her wounds were dressed and she was referred to Wardha civil hospital the same day. Here, investigations were done with computed tomography (CT) scan and X-ray. She regained consciousness after three to four hours and was able to recognize the people around her. The CT scan reports were normal and an X-ray showed a tri-malleolar fracture of the right ankle with ankle subluxation. Open reduction and internal fixation were done to manage the fracture, and a plaster cast was given for one-and-a-half months from mid-thigh to mid-foot. She was discharged from the hospital and was followed up after 15 days. After removing the cast, it was observed that the sutures had not healed properly and so she was called for dressing every eight days.

Around two months after the operation, she saw pus discharge from the unhealed wound and so she was advised to undergo skin grafting. She was hospitalized for 15 days during which time she was given injections and medications and the wound showed improvement in healing so grafting was not done. She was advised to be non-weight-bearing during this time. Again, six months later, she saw pus discharge from a small opening on the operation site. An X-ray showed bone healing so implant removal was advised. She was operated on for implant removal. After this operation, her sutures healed properly and she was advised weight-bearing on the leg after one-and-a-half months. But due to the fear of pain and complications, the patient refrained from placing weight on the injured ankle while walking. She walked with the help of a walker for two months post-operatively, after which, a tripod stick was advised by the doctor. She was not on any medication and had no trauma during this post-operative period. As she was keen to rejoin her job, she undertook physiotherapy at the outpatient department (OPD) six months after implant removal with the chief complaint of difficulty in walking.

Clinical findings

Before performing the physical examination, the patient's verbal and written consent was obtained. Dull aching pain on the operative site was 4/10 on movement while weight-bearing on the right ankle and 0/10 on rest according to the numerical pain rating scale (NPRS). Mild swelling and grade 2 tenderness were present on palpation around the scar. A 10cm scar was present on the medial aspect of the leg over the medial malleolus. The borders of the scar were hypopigmented and the center was hyperpigmented, and the pliability was yielding on palpation. The height of the scar was less than 2mm. Muscle-wasting was seen over the calf muscles of the right leg which was confirmed by girth measurement that showed a 2cm difference. A true leg length discrepancy of 1cm was seen in the right leg. The active and passive movements of the right ankle were painful and restricted. The right foot was fixed in a plantarflexion position. The gait was altered as walking with the assistance of a tripod stick resulted in a right pelvic hike with an absent heel strike, reduced knee flexion throughout the swing phase, and reduced stance phase of the right limb. Step length was 20cm, stride length was 41cm, step width was 15cm and cadence was 77 steps per min. 

Radiological investigations

**Figure 1:**
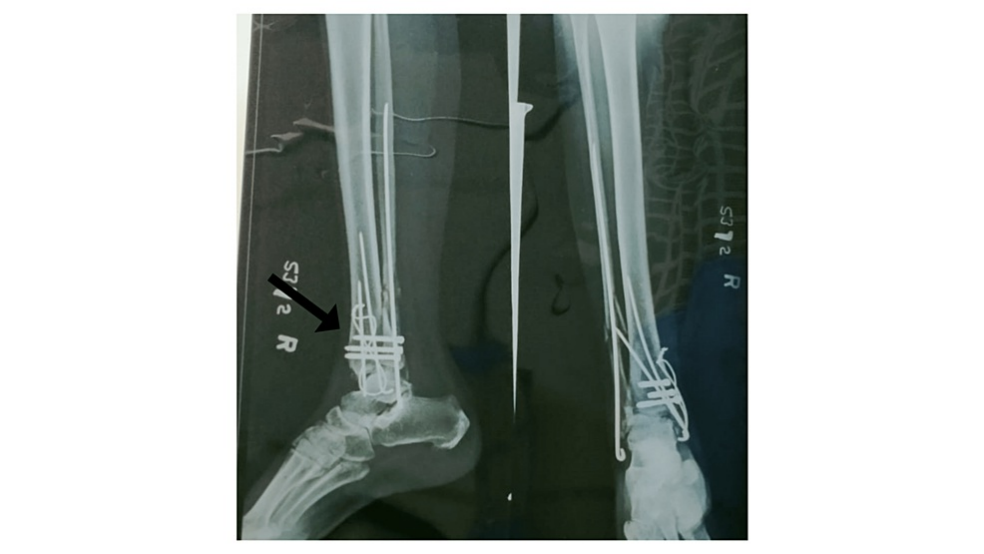
Post-operative X-ray of the right ankle in anteroposterior (AP) view and lateral view showing the trimalleolar fracture (black arrow) managed with open reduction and internal fixation using a rush nail and cannulated cancellous screw.

The radiological investigations were conducted post-operatively to view the fixation of the implants (Figure 1).

Surgical procedure

In the first operation, open reduction and internal fixation were done. Cleaning, painting, and draping were performed successfully with all aseptic precautions. Fibula and medial malleolus were exposed through the medial aspect. The distal one-third tibia with posterior malleolus plus medial malleolus was reduced by rush nailing and cannulated cancellous (CC) screw with tension band wiring.

The second operation was done for implant removal. Aseptic precautions were taken for cleaning, painting, and draping. Through an incision given over the previous scar, the CC screw and rush nail were removed completely.

Therapeutic intervention

When the patient was followed-up at the physiotherapy OPD after six months of implant removal, the goals were to reduce pain, improve the range of motion of the right ankle, improve the strength of the lower extremity, and improve the scar pliability, proprioception training, and gait training. 

Mild swelling was present only over the medial malleolus so it was treated with cryotherapy for 10 minutes. The pain in the calf muscle of the right leg that resulted while walking, was treated with a hot pack for 15 minutes. This helped increase blood flow to the area and remove metabolic waste, thus blocking the pain perceptions and reducing pain to help relax the muscle.

**Figure 2:**
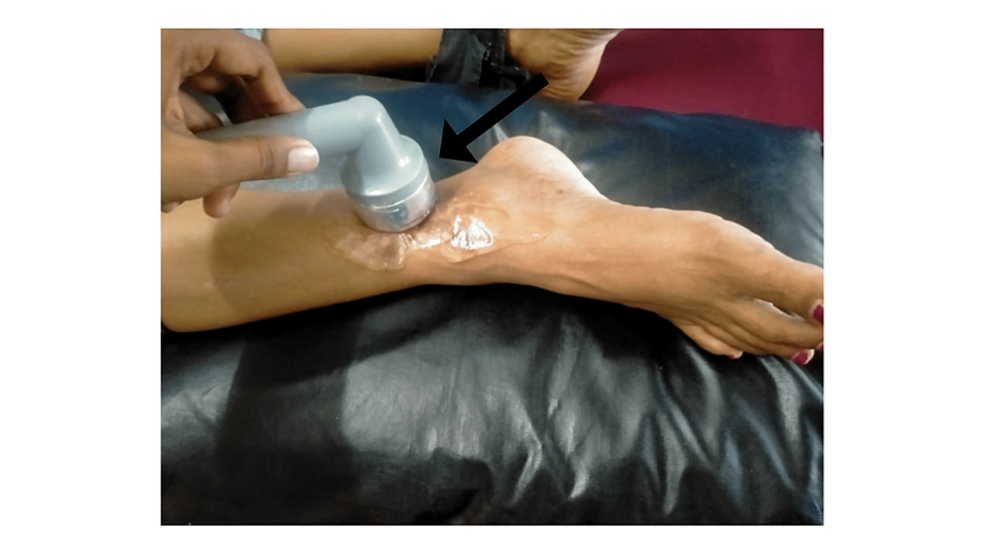
Scar being treated with ultrasound

Ultrasound was given over the scar tissue to increase pliability (Figure 2). The continuous mode was used, with 1watt/cm2 intensity for seven minutes. The scar was treated with a cross-friction massage using a gel for 10 minutes.

**Figure 3:**
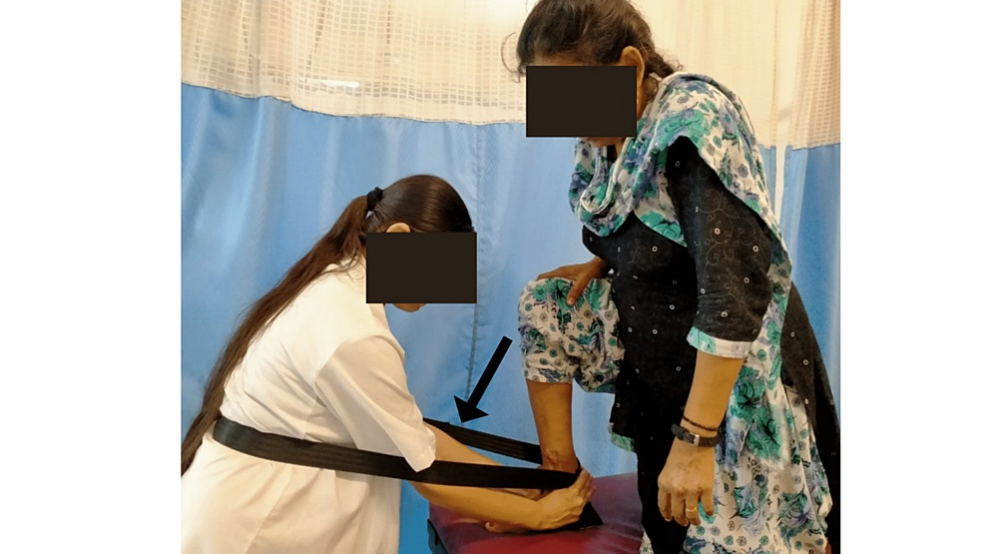
The Mulligan's belt used for movement and mobilization to increase dorsiflexion

Movement with mobilization was given to increase dorsiflexion using a Mulligan’s belt (Figure 3). The patient was placed in a half-kneeling position with weight-bearing on the affected right foot. Facing the patient in a standing position the mobilization belt was placed around the physiotherapist's lower back and the patient's Achilles tendon. A palm was placed on the foot's dorsum and the web spaces of the hands around the talus neck. The subtalar joint was kept neutral and the foot was firmly planted. The belt at the ankle joint produced a pain-free, graded anterior gliding force. The patient was told to lunge forward while maintaining this position, which aided painless end-range loading and dorsiflexion of the injured ankle. This routine was repeated for three sets.

The patient's tendon Achilles was stretched with the patient in a long sitting position holding a non-elastic band in both hands which were placed around the plantar aspect of the foot. The stretch into dorsiflexion was held for 10 seconds and repeated 10 times.

**Figure 4:**
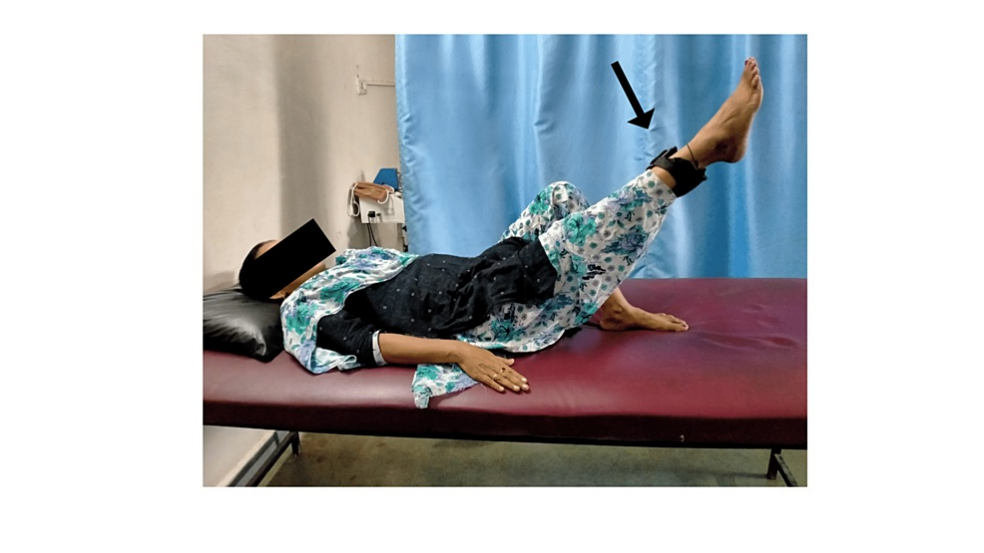
Strength training of hip flexors using a 1kg weight cuff

**Figure 5:**
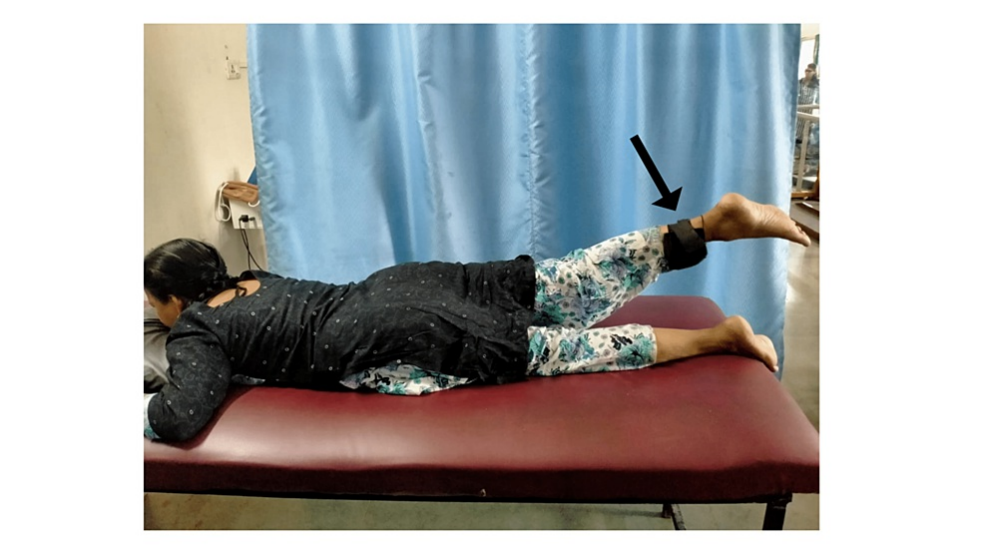
Strength training of hip extensors in prone lying position using a 1kg weight cuff

Hip muscle strengthening was done with a 1kg weight cuff. For hip flexors i.e., iliopsoas muscle, the patient was placed in a supine position (Figure 4), extensors i.e., gluteus Maximus were in a prone lying position (Figure 5), and abductors i.e., gluteus medius and minimus were in a side-lying position. Ten repetitions of each were given. Dynamic quadriceps strengthening was given in a high sitting position with a 1kg weight cuff 10 times. Strengthening of the dorsiflexors and plantar flexors was done using the resistance band, 10 times each. Progression was done to heel raise and toe raise in standing for 10 times.

Proprioception training was given to improve weight-bearing on the right foot, weight shifts, step up and step down, taking weight on the right limb, and forward flexion, extension, and abduction of the left leg. Ten repetitions of each were done. Perturbations with a stick were given in different directions to maintain balance.

Gait training in the parallel bar was also done. Instructions were given to place the foot first with a heel strike while walking, and knee flexion while placing the foot forward. Shoe raise was advised to the patient.

Follow-up and outcome measure

The follow-up was done after the third week of physiotherapy. Table 1 shows the pre and post-treatment values of different parameters.

**Table 1:** The before and after treatment values of different outcomes

	Right leg Day 1 (6/6/22)	Right leg Day 21 (27/6/22)
Pain on numerical pain rating scale		
During weight-bearing on the right foot	4/10	2/10
Ankle range of motion (in degrees)		
Dorsiflexion	0-5	0-12
Plantar-flexion	0-20	0-25
Manual muscle testing (MMT)		
Hip		
Flexors	3+/5	4/5
Extensors	3+/5	4/5
Abductors	3+/5	4/5
Adductors	3+/5	4/5
Ankle		
Dorsiflexors	3-/5	3/5
Planta-flexors	3-/5	3/5
Leg-length discrepancy		
Distance of heel from the floor in stance standing	4cm	1.5cm
Tenderness		
Around the scar	Grade 2	Grade 1
Gait parameters		
Step length	20cm	25cm
Step width	15cm	18cm
Stride length	41cm	51cm
Cadence	77steps/min	85steps/min

## Discussion

To absorb body weight during jump landings, gait deceleration, and eccentric motions, ankle dorsiflexion (DF) range of motion (ROM) is important. In the closed kinetic chain (CKC), the anterior sliding of the tibia over the talus and the posterior sliding of the talus over the tibia is responsible for the arthro-kinematic movement of the DF. When doing CKC activities, DF restriction may make it difficult for the tibia to move over the talus, restrict knee flexion, reduce the capacity of the body to withstand eccentric loads, and cause the body to make compensatory movements with the knee and hip in the frontal plane [7]. After surgery, ankle edema is a typical and long-lasting complication. As per a report, stiffness, swelling, and pain were reported by more than half of the patients who had unimalleolar and bimalleolar ankle fractures. One year after ankle fractures, 60% or more of patients 65 years of age and older reported ankle pain, swelling, and problems using stairs, as well as a decrease in daily activities [8]. With interactions at the local, central nervous system, and psychological levels, manual therapy such as joint mobilization causes an analgesic effect and increases the flexibility of joint structures [9]. According to reports, postural stability, coordination, and proprioception may all benefit from balance and proprioception training included during the rehabilitation program after an ankle injury [1]. In this case report, a patient came for physiotherapy 15 months after the fracture due to complications after an operation. Due to this gap in time, the range that could be achieved at its maximum previously cannot be achieved now. After the increase in some degrees of dorsiflexion by Mulligan's mobilization and exercise program, a shoe raise was advised to the patient so that her functional limitations can be overcome. 

## Conclusions

In this case report, we describe the three weeks of physiotherapy management for chronic post-operative trimalleolar ankle fracture. Physiotherapy management which included Mulligan’s movement with mobilization, ultrasound therapy, strength training, proprioceptive training, and gait training, showed improvement in range and strength of ankle dorsiflexion, and overall functioning of the patient. 
